# Unravelling the effects of age, period and cohort on metabolic syndrome components in a Taiwanese population using partial least squares regression

**DOI:** 10.1186/1471-2288-11-82

**Published:** 2011-05-27

**Authors:** Yu-Kang Tu, Kuo-Liong Chien, Victoria Burley, Mark S Gilthorpe

**Affiliations:** 1 Division of Biostatistics, Centre for Epidemiology and Biostatistics, University of Leeds, Leeds, UK; 2 Leeds Dental Institute, University of Leeds, Leeds, UK; 3 Institute of Preventive Medicine, College of Public Health, National Taiwan University, Taipei, Taiwan; 4 Nutrition Epidemiology Group, School of Food Science and Nutrition, University of Leeds, Leeds, UK

**Keywords:** Metabolic syndrome, obesity, age-period-cohort analysis, partial least squares, Taiwan

## Abstract

**Background:**

We investigate whether the changing environment caused by rapid economic growth yielded differential effects for successive Taiwanese generations on 8 components of metabolic syndrome (MetS): body mass index (BMI), systolic blood pressure (SBP), diastolic blood pressure (DBP), fasting plasma glucose (FPG), triglycerides (TG), high-density lipoprotein (HDL), Low-density lipoproteins (LDL) and uric acid (UA).

**Methods:**

To assess the impact of age, birth year and year of examination on MetS components, we used partial least squares regression to analyze data collected by Mei-Jaw clinics in Taiwan in years 1996 and 2006. Confounders, such as the number of years in formal education, alcohol intake, smoking history status, and betel-nut chewing were adjusted for.

**Results:**

As the age of individuals increased, the values of components generally increased except for UA. Men born after 1970 had lower FPG, lower BMI, lower DBP, lower TG, Lower LDL and greater HDL; women born after 1970 had lower BMI, lower DBP, lower TG, Lower LDL and greater HDL and UA. There is a similar pattern between the trend in levels of metabolic syndrome components against birth year of birth and economic growth in Taiwan.

**Conclusions:**

We found cohort effects in some MetS components, suggesting associations between the changing environment and health outcomes in later life. This ecological association is worthy of further investigation.

## Background

Several Asian countries have achieved great economic growth in the second half of the last century and experienced rapid industrialization, urbanization and social change. As a result, living environments in these countries have gone through a dramatic transformation, and people started to adopt, gradually, western lifestyles and food. The potential consequences and impacts of this westernization on general health in the populations within these newly developed countries have been much discussed in the literature, especially around the increased prevalence of obesity giving rise to greater risks of chronic diseases, such as type-2 diabetes and certain types of cancers thought to be related to western diets [1-3].

Moreover, according to the developmental origins of health and disease hypothesis [4-6], for people in countries who were born before or at the start of rapid economic growth, there may be an increased risk of developing chronic adult diseases due to a mismatch between early and later environments. Those generations born after economic growth has achieved a certain level of national wealth may, however, benefit from better neonatal and postnatal nutrition and medical care [7-9], thereby yielding better health outcomes in adult life than observed for previous generations. While the problems of obesity and related chronic diseases should not be overlooked [10-13], the increased wealth and improved living standard due to economic growth may exert a protective effect on health in later life [4-6].

Many studies have investigated the adverse impact of changes in dietary patterns and lifestyles on the population health in newly developed Asian countries such as South Korea, Taiwan and Hong Kong [1,3,10-13], but the potential beneficial impact of improved early nutrition is still not well understood. For instance, Taiwan has a population of over 23 million; whilst Taiwanese economy has been growing steadily since the end of the Second World War, it experienced spectacular economic growth during the last three decades, and the living environment (e.g. increased affordability and availability of food, transportation, housing etc.) has been transformed dramatically. Several studies have reported that the prevalence of obesity has been increasing [14-16]. As obesity is shown to be associated with an increased risk of cardiovascular diseases and diabetes in adults, it is anticipated that the prevalence of obesity related diseases in the Taiwanese population will also increase [17-21].

The rapid economic and social transformations may have differential impacts across generations who were born and raised in different stages of economic development in Taiwan [5,6,22]. Whilst many studies have shown an increased prevalence of obesity and the potential public health implications, it remains to be ascertained that the risks of chronic diseases have also been growing across generations. The aim of this study is to use health screening data collected in Taiwan in the years 1996 and 2006 to disentangle the counteractive effects on health caused by economic growth and social change by undertaking an age-period-cohort analysis on components of metabolic syndrome.

## Methods

### Mei-Jaw health screening data

Mei-Jaw (MJ) Corporation is a Taiwanese private organization providing health screening services for its members. Details of the MJ Health Screening scheme and data collection have been described elsewhere [23,24]. Data collected by its four clinics in Taiwan were computerized from 1994 onwards and questionnaires about personal and medical histories, lifestyles and diets were collected from 1996. Weight and height were measured by an auto-anthropometer, Nakamura KN-5000A (Nakamura, Tokyo, Japan). Weight was measured to the nearest 0.1 kg with subjects standing barefoot and wearing light indoor clothing. Height was recorded to the nearest 0.1 cm. BMI was calculated as body weight divided by height (in meters) squared and used as a proxy variable for obesity. Overnight fasting blood was collected and analyzed (Hitachi 7150 auto-analyzers, Tokyo, Japan). In addition to BMI, seven other components of the metabolic syndrome are investigated in this study: fasting plasma glucose (FPG), triglycerides (TG), high-density lipoprotein cholesterol (HDL), low-density lipoprotein cholesterol (LDL), uric acid (UA), and systolic and diastolic blood pressure. For the latter, the mean of 2 seated measurements taken at 10 minute intervals using a computerized auto-mercury-sphygmomanometer were used Citizen CH-5000 (Citizen, Tokyo, Japan). To reduce potential biases caused by the use of medicines, only people between 20 and 59 years, who reported no history of chronic diseases such as diabetes, hypertension, and cancer, and who were not on long-term medication, were included in the analysis: 14,362 subjects from the 71,233 examined in 1996 (20%), and 28,524 subjects from the 80,851 (35%) examined in 2006.

### Ethics

Written consent for using the screening results for academic research was obtained from each participant, when she/he attended the clinics for health screening, and this research project has been approved by the Research Ethics Committee at the University of Leeds.

### Statistical analysis

Sex and year-specific adjusted values of the eight metabolic syndrome components were first obtained by using linear regression with adjustment for years in formal education, history and frequency of cigarettes smoking, alcohol intake and betel-quid chewing. An age-period-cohort analysis was then undertaken separately for men and women, combining the data from 1996 and 2006. As age at examination (between 20 and 59), time period (examination year 1996 or 2006), and cohort (year of birth between 1937 and 1986) are mathematically related (time period = age at examination + cohort), it is well known that these three variables cannot be entered into the same regression model due to perfect collinearity. To resolve this problem, we used partial least squares regression (PLSR) to separate the effects of age, period and cohort.

PLSR is a statistical methodology widely used in chemometrics and bioinformatics for the analysis of data with the number of variables exceeding the number of observations [25-28]. Similar to principal component analysis (PCA), PLSR extracts components that are a weighted combination of the original variables; however, whilst PCA aims to maximize the variances of successively extracted components, i.e. the variance of the first component is larger than that of the second, and the second is larger than the third etc., under the constraints that all the components are independent and the sum of the squared weights is unity, PLSR aims to maximize the covariance between the outcome and successively extracted components, i.e. the covariance between the outcome and the first component is larger than that between the outcome and the second, and the covariance between the outcome and the second component is larger than the covariance between the outcome and the third etc., under the same two constraints. This process leads to a unique partition of covariance between the outcome and all covariates. Usually, the first few components can explain most of the covariance with the outcome, and this can substantially reduce the problem of unstable regression coefficients caused by collinearity.

One advantage of PLSR over traditional regression analysis is that perfectly collinear variables can be considered simultaneously as covariates in one model, i.e. all the three variables, age at examination, time period, and cohort can be entertained into the same model. Consequently, it becomes possible to estimate their individual effects on the outcomes. This is because PLS does not use the original collinear covariates in the computational process; instead, it constructs weighted component first and then maximizes the covariance between the outcome and those weighted components successively extracted under the two constraints that all the components are independent and the sum of the squared weights is unity. Those constraints ensure that the weights, and therefore the regression coefficients for the covariates, are unique and maximize the covariance extraction in the process [25-29]. A more detailed and technical explanation for how PLSR resolves the identification issue is provided in the appendix.

The use of PLSR aims to develop parsimonious models with the first few components only, hence increments in the explained variance in the outcome (e.g. changes in *R*^2^) are used as a criterion for selecting the number of PLSR components. This provides a measure of predictive ability, the predictive residual error sum of squares (PRESS) [29]. To obtain this, the data are first split into a number of groups. For each, a prediction is obtained using the model derived from all other groups. For example, one observation is left out of the model, and we use the remaining observations to predict the outcome. PRESS is calculated as the sum of squares of the differences between the prediction for each observation (when it is left out of the model) and the observed value of the dependent variables.

We first tested linear relations between the outcomes (each of the eight metabolic syndrome components) and age at examinations (*age*) and cohort, i.e. year of birth (*birthyr*). To improve stability in the iteration process for PLSR, age was centred on 20 years and *birthyr *was centred on 1937. The variable for the period effect, age at examination (*examyr*), was a binary variable (1996 code 0, 2006 coded 1). To explore nonlinear relationships, we constructed restricted cubic splines for *age *and *birthyr *with five knots at ages 24, 30, 35, 41, and 53 years for *age *and at years 1949, 1961, 1968, 1973, and 1980 for *birthyr. *These knots represent the 0.05, 0.275, 0.5, 0.725 and 0.95 percentiles within each variable, as suggested by Harrell [30].

Subjects with missing values (a few hundred for LDL and HDL in 2006, and very few for the other variables; see Tables 1 and 2 and Additional File 1 for greater detail) were excluded from data analysis. Statistical analyses were undertaken using the statistical software packages STATA (version 11.1, StataCorp, Texas, USA) and XLSTAT (version 2009.6.04, Addinsoft). Confidence intervals for PLSR coefficients were obtained using the jack-knife method, because there is no distribution assumption for PLSR coefficients [29].

**Table 1:** Body size, components of the metabolic syndrome, and aspects of lifestyle in 1996 and 2006 for men.

		Total			20-29			30-39			40-49			50-59	
	
	N	Mean	SD	N	Mean	SD	N	Mean	SD	N	Mean	SD	N	Mean	SD
Year 1996															
Body weight (kg)	6520	68.00	10.38	1940	66.70	10.97	2762	68.82	10.63	1192	69.10	9.23	626	66.35	8.69
Body height (cm)	6520	169.58	6.00	1940	171.04	5.97	2762	169.96	5.83	1192	168.42	5.60	626	165.56	5.42
BMI (kg/m^2^)	6520	23.63	3.22	1940	22.77	3.33	2762	23.80	3.25	1192	24.34	2.88	626	24.19	2.77
FPG (mg/dl)	6520	96.87	14.51	1940	94.13	11.68	2762	96.26	11.10	1192	99.76	17.30	626	102.52	24.21
SBP (mmHg)	6520	117.99	14.66	1940	117.87	13.83	2762	116.80	13.67	1192	118.08	15.23	626	123.46	18.55
DBP (mmHg)	6520	73.07	9.83	1940	71.06	9.29	2762	72.84	9.51	1192	75.26	10.32	626	76.11	10.36
TG (mg/dl)	6520	131.81	104.38	1940	106.65	79.54	2762	140.33	110.73	1192	148.19	111.83	626	140.95	114.63
HDL (mg/dl)	6519	41.27	12.18	1940	40.93	11.19	2762	41.04	12.49	1192	41.50	12.34	626	42.88	13.29
LDL (mg/dl)	6401	125.52	31.31	1924	116.94	28.45	2695	125.79	30.62	1165	134.24	32.52	617	134.60	33.18
UA (mg/dl)	6520	6.88	1.36	1940	6.93	1.36	2762	6.95	1.37	1192	6.81	1.32	626	6.60	1.37

Year 2006															
Body weight (kg)	14261	70.76	10.79	3109	70.54	11.89	6292	71.55	10.90	3538	70.37	9.82	1322	68.53	9.53
Body height (cm)	14261	171.56	6.08	3109	173.02	5.88	6292	172.21	5.88	3538	170.52	5.90	1322	167.76	5.93
BMI (kg/m^2^)	14261	24.01	3.24	3109	23.54	3.65	6292	24.10	3.25	3538	24.18	2.94	1322	24.31	2.83
FPG (mg/dl)	14258	98.55	14.31	3109	94.80	9.07	6289	97.58	12.18	3538	101.42	17.90	1322	104.25	18.97
SBP (mmHg)	14259	119.72	13.69	3109	120.10	12.61	6291	119.19	13.12	3537	119.22	14.40	1322	122.65	16.22
DBP (mmHg)	14259	71.38	9.95	3109	68.76	9.02	6291	71.02	9.42	3537	72.98	10.47	1322	74.98	11.18
TG (mg/dl)	14259	128.30	98.03	3109	97.39	61.81	6290	131.77	92.44	3538	146.24	124.72	1322	136.44	95.74
HDL (mg/dl)	13300	48.05	10.89	2918	49.69	10.70	5843	47.30	10.77	3269	47.60	10.96	1270	48.93	11.34
LDL (mg/dl)	13294	122.29	30.96	2917	112.86	28.70	5837	122.56	30.32	3269	127.75	31.80	1271	128.66	31.56
UA (mg/dl)	14250	6.56	1.23	3109	6.68	1.24	6288	6.62	1.23	3532	6.41	1.20	1321	6.35	1.21

A more detailed version is provided in the online additional file 1. Abbreviation: body mass index (BMI), systolic blood pressure (SBP), diastolic blood pressure (DBP), fasting plasma glucose (FPG), triglycerides (TG), high-density lipoprotein (HDL), Low-density lipoproteins (LDL), uric acid (UA).

**Table 2:** Body size, components of the metabolic syndrome, and aspects of lifestyle in 1996 and 2006 for women.

		Total			20-29			30-39			40-49			50-59	
	
	N	Mean	SD	N	Mean	SD	N	Mean	SD	N	Mean	SD	N	Mean	SD
Year 1996															
Body weight (kg)	7841	54.68	8.15	2534	52.75	8.09	3102	54.30	7.50	1368	57.36	8.47	837	57.59	8.15
Body height (cm)	7841	157.46	5.36	2534	158.85	5.29	3102	157.84	5.11	1368	156.30	4.93	837	153.71	5.07
BMI (kg/m^2^)	7841	22.07	3.23	2534	20.90	3.00	3102	21.80	2.85	1368	23.47	3.24	837	24.36	3.17
FPG (mg/dl)	7841	93.64	12.23	2534	90.94	8.85	3102	92.79	9.31	1368	95.96	14.84	837	101.13	19.82
SBP (mmHg)	7841	111.16	15.41	2534	107.65	12.43	3102	107.96	12.98	1368	115.52	16.62	837	126.56	18.41
DBP (mmHg)	7841	69.32	9.62	2534	67.43	8.86	3102	68.11	8.98	1368	71.66	9.94	837	75.67	10.19
TG (mg/dl)	7841	87.77	58.81	2534	75.57	34.80	3102	83.70	48.70	1368	98.23	64.77	837	122.69	105.81
HDL (mg/dl)	7841	49.06	12.63	2534	49.07	12.38	3102	48.68	12.34	1368	49.13	13.11	837	50.38	13.59
LDL (mg/dl)	7819	119.96	30.36	2533	112.13	27.61	3095	117.94	27.74	1361	125.53	30.07	830	142.26	35.46
UA (mg/dl)	7841	5.08	1.16	2534	5.05	1.10	3102	5.00	1.11	1368	5.05	1.21	837	5.47	1.33

Year 2006															
Body weight (kg)	14259	54.28	8.52	3335	52.76	9.07	5942	54.06	8.53	3289	55.21	7.92	1693	56.23	7.85
Body height (cm)	14259	158.86	5.42	3335	160.11	5.27	5942	159.57	5.16	3289	158.02	5.31	1693	155.52	5.25
BMI (kg/m^2^)	14259	21.51	3.23	3335	20.57	3.30	5942	21.22	3.12	3289	22.11	2.98	1693	23.25	3.03
FPG (mg/dl)	14259	93.54	11.06	3334	90.34	6.77	5943	92.43	9.81	3289	95.26	11.01	1693	100.39	17.03
SBP (mmHg)	14253	109.59	14.24	3333	106.55	11.73	5939	107.02	12.24	3289	111.65	14.74	1692	120.61	17.86
DBP (mmHg)	14253	64.11	9.63	3333	61.78	8.19	5939	63.03	8.74	3289	65.45	10.22	1692	69.87	11.26
TG (mg/dl)	14259	79.44	51.68	3334	65.37	32.21	5943	74.94	42.94	3289	86.24	55.08	1693	109.72	81.37
HDL (mg/dl)	13541	59.86	13.44	3060	60.84	13.56	5909	59.62	13.37	3197	59.29	13.16	1675	59.98	13.88
LDL (mg/dl)	13539	111.92	29.29	3059	103.04	26.16	5608	108.56	26.96	3198	115.66	28.31	1674	132.28	33.14
UA (mg/dl)	14258	4.66	1.01	3334	4.67	0.98	5943	4.59	0.97	3289	4.57	0.99	1692	5.04	1.12

A more detailed version is provided in the online additional file 1. Abbreviation: body mass index (BMI), systolic blood pressure (SBP), diastolic blood pressure (DBP), fasting plasma glucose (FPG), triglycerides (TG), high-density lipoprotein (HDL), Low-density lipoproteins (LDL), uric acid (UA).

## Results

### Comparison of Variables between 1996 and 2006

Tables 1 and 2 provide summary statistics for the eight metabolic syndrome components and the anthropometric variables for subjects examined in 1996 and 2006 for males and females, respectively. On average, men had greater weight, height, BMI, SBP, FPG, but lower DBP, lower TG, lower LDL, lower UA and higher HDL in 2006 than in 1996. On average, women were slightly taller in 2006 than in 1996, but they had a similar weight, giving rise to a smaller BMI. For women, SBP, DBP, TG, LDL and UA were lower and HDL was higher in 2006, whilst FPG remained at a similar level. Men had higher BMI, FPG, SBP, DBP, TG, LDL and UA but lower HDL than women in both 1996 and 2006.

Younger participants tended to have higher educational attainment, and on average participants in 2006 had spent longer in formal education than those in 1996. Within each of the four age groups, men were heavier and taller in 2006 than in 1996, as too were women.

**Figure 1:**
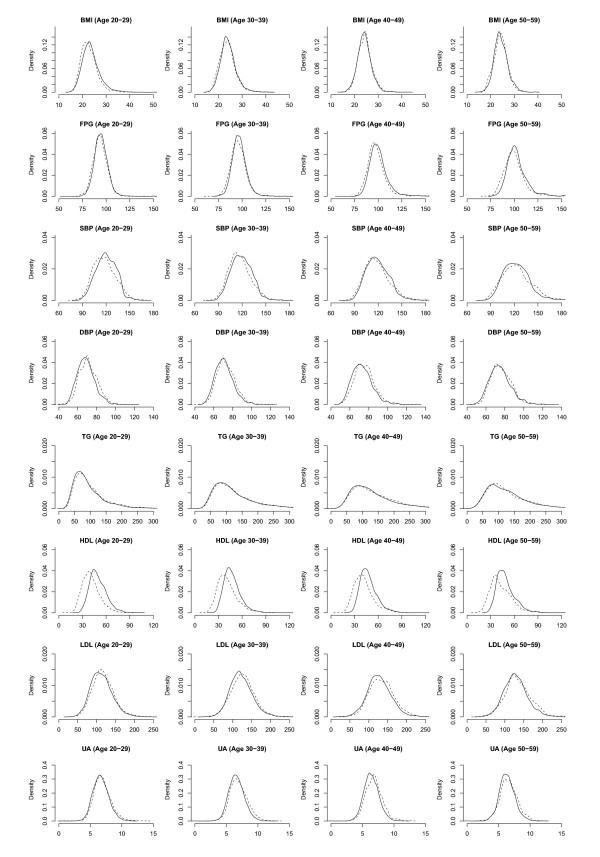
**Kernel density plots of body mass index (BMI), fasting plasma glucose (FPG), systolic blood pressure (SBP), diastolic blood pressure (DBP), triglycerides (TG) and high-density lipoproteins (HDL) for men in 1996 (dashed line) and 2006 (solid line)**.

**Figure 2:**
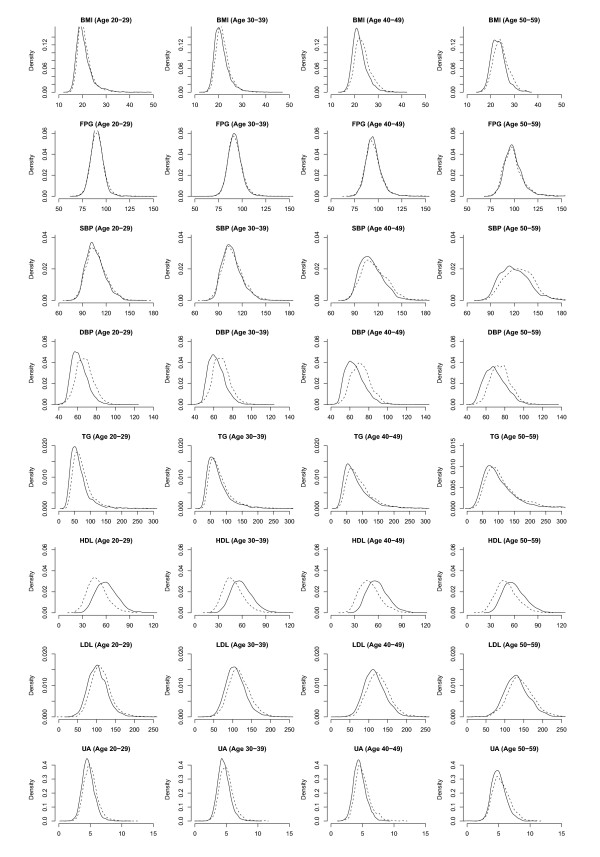
**Kernel density plots of body mass index (BMI), fasting plasma glucose (FPG), systolic blood pressure (SBP), diastolic blood pressure (DBP), triglycerides (TG) and high-density lipoproteins (HDL) for women in 1996 (dashed line) and 2006 (solid line)**.

The kernel density plots of the eight metabolic syndrome components for men in Figure 1 show that there was little difference in the distributions of BMI, FPG, and TG between 1996 and 2006. The distributions of SBP slightly shifted to the right in the younger age groups in 2006, indicating a small increase in the mean, whilst DBP slightly shifted to the left, showing a small decrease in the mean. The distribution of HDL, however, shifted to the right in 2006 for all age groups, indicating that men had a higher HDL in 2006. The distributions of LDL and UA shifted to the left. The kernel density plots in Figure 2 for women showed that the distributions of BMI and DBP shifted slightly to the left, indicating a small decrease in the means. There was also a left shift in distribution of TG, LDL and UA, though a right shift in HDL.

### Age-period-cohort analysis

**Figure 3:**
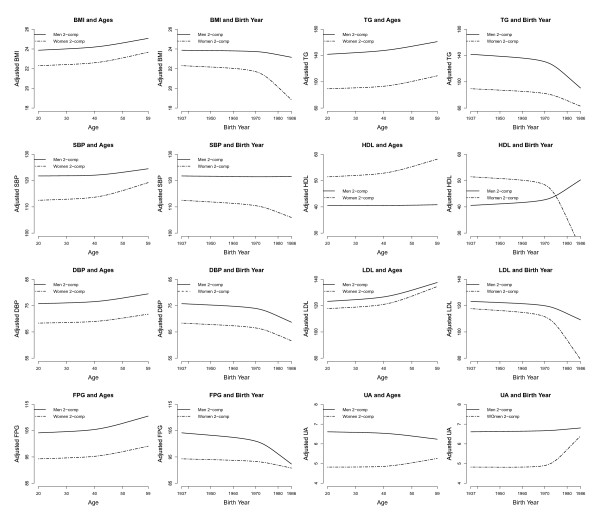
**Associations between adjusted values of components of metabolic syndrome and age at examinations/birth year**. Confounders adjusted for were years in formal education, history and frequency of cigarettes smoking, alcohol intake and betel-quid chewing. Results are from partial least squares regression models with 2 components.

Tables 3 showed the results of the linear PLSR, and Figure 3 and Table 4 showed the results from nonlinear (restricted cubic splines) PLSR. PRESS, the statistical index for the selection of components, selected only the first PLS component for all linear or nonlinear models. Nevertheless, to examine the robustness of our findings, we also look at results from models with the first two components are also presented. In general, the one component model explained more than 75% of the total *R*^2^, and the two component models explained more than 95% of the total *R*^2^. The greater the *R*^2 ^in the one-component models, the smaller the differences caused by adding the second component to the models. As the two-component models explained almost all the total *R*^2^, adding more components yields little difference to results, i.e. insubstantial changes to the estimated regression coefficients. Consequently, we concentrated on the interpretation of results from two-component models.

**Table 3:** Results from linear partial least squares regression analysis with 2 components: *Exam year *is a binary variable [1996 coded 0, 2006 coded 1); *Birth year *is centered at 1937; *Age *is centered at 20 years.

		**Men**		**Women**	
		
**BMI**					
	Birth year	0.00	(-0.02 to 0.01)	-0.03	(-0.04 to -0.03)
	Exam year	0.93	(0.09 to 1.77)	-0.93	(-1.18 to -0.68)
	Age	0.03	(0.01 to 0.04)	0.02	(0.01 to 0.02)
**SBP**					
	Birth year	-0.02	(-0.05 to 0.01)	-0.14	(-0.18 to -0.11)
	Exam year	-0.49	(-2.00 to 1.03)	-3.14	(-4.89 to -1.40)
	Age	0.01	(-0.01 to 0.02)	0.09	(0.05 to 0.12)
**DBP**					
	Birth year	-0.11	(-0.13 to -0.09)	-0.11	(-0.13 to -0.09)
	Exam year	-2.96	(-4.02 to -1.9)	-4.12	(-5.37 to -2.87)
	Age	0.05	(0.02 to 0.08)	0.02	(-0.02 to 0.05)
**FPG**					
	Birth year	-0.19	(-0.23 to -0.15)	-0.06	(-0.09 to -0.04)
	Exam year	-5.84	(-7.72 to -3.95)	1.81	(0.53 to 3.09)
	Age	0.07	(0.01 to 0.12)	0.12	(0.09 to 0.15)
**TG**					
	Birth year	-0.60	(-0.87 to -0.32)	-0.49	(-0.56 to -0.42)
	Exam year	-2.08	(-21.7 to 17.55)	-9.58	(-12.71 to -6.45)
	Age	0.66	(0.41 to 0.92)	0.32	(0.22 to 0.43)
**HDL**					
	Birth year	0.08	(0.05 to 0.10)	0.07	(0 to 0.14)
	Exam year	4.97	(2.60 to 7.33)	3.25	(0.24 to 6.27)
	Age	0.05	(-0.01 to 0.10)	0.01	(-0.03 to 0.04)
**LDL**					
	Birth year	-0.17	(-0.27 to -0.06)	-0.32	(-0.39 to -0.25)
	Exam year	8.71	(-24.51 to 41.92)	-0.44	(-24.86 to 23.99)
	Age	0.45	(-0.42 to 1.32)	0.32	(0.25 to 0.39)
**UA**					
	Birth year	0.00	(0.00 to 0.01)	0.00	(0.00 to 0.00)
	Exam year	-0.20	(-0.29 to -0.11)	-0.27	(-0.37 to -0.17)
	Age	-0.01	(-0.01 to -0.01)	0.00	(0.00 to 0.00)

Abbreviation: body mass index (BMI), systolic blood pressure (SBP), diastolic blood pressure (DBP), fasting plasma glucose (FPG), triglycerides (TG), high-density lipoprotein (HDL), Low-density lipoproteins (LDL), uric acid (UA).

**Table 4:** Results from restricted cubic splines partial least squares regression analysis with 1 or 2 components: *Exam year *is a dummy variable (1996 coded 0 versus 2006 coded 1); restricted cubic splines create 4 variables for each of Ages and Birthyr.

	Men (1-comp)		Men (2-comp)		Women (1-comp)		Women (2-comp)	
	
	Coef	95%CIs	Coef	95%CIs	Coef	95%CIs	Coef	95%CIs
FPG								
Exam year	-1.45	(-2.14 to -0.75)	-4.24	(-5.26 to -3.23)	0.28	(0.00 to 0.56)	1.37	(0.54 to 2.21)
Ages_1	0.05	(0.03 to 0.06)	0.00	(-0.05 to 0.04)	0.03	(0.03 to 0.04)	0.05	(0.03 to 0.07)
Ages_2	0.05	(0.03 to 0.06)	0.00	(-0.03 to 0.03)	0.03	(0.03 to 0.04)	0.04	(0.03 to 0.05)
Ages_3	0.10	(0.06 to 0.14)	0.00	(-0.06 to 0.05)	0.07	(0.06 to 0.09)	0.09	(0.07 to 0.11)
Ages_4	0.24	(0.12 to 0.37)	-0.03	(-0.14 to 0.08)	0.18	(0.15 to 0.22)	0.22	(0.16 to 0.27)
Birthyr_1	-0.07	(-0.10 to -0.05)	-0.10	(-0.14 to -0.05)	-0.02	(-0.03 to -0.02)	-0.01	(-0.02 to 0.00)
Birthyr_2	-0.07	(-0.09 to -0.04)	-0.09	(-0.11 to -0.06)	-0.02	(-0.03 to -0.01)	-0.01	(-0.03 to 0.00)
Birthyr_3	-0.31	(-0.45 to -0.18)	-0.38	(-0.55 to -0.20)	-0.09	(-0.13 to -0.05)	-0.04	(-0.12 to 0.04)
Birthyr_4	-1.18	(-1.76 to -0.60)	-1.29	(-2.24 to -0.34)	-0.32	(-0.50 to -0.14)	-0.09	(-0.42 to 0.24)

SBP								
Exam year	-0.18	(-0.43 to 0.07)	-0.51	(-1.39 to 0.37)	-0.76	(-1.19 to -0.33)	-3.85	(-5.99 to -1.70)
Ages_1	0.01	(-0.03 to 0.05)	-0.01	(-0.08 to 0.06)	0.04	(0.03 to 0.04)	-0.01	(-0.05 to 0.04)
Ages_2	0.02	(-0.04 to 0.08)	0.03	(-0.06 to 0.11)	0.05	(0.04 to 0.06)	0.06	(0.04 to 0.09)
Ages_3	0.05	(-0.08 to 0.19)	0.09	(-0.09 to 0.26)	0.11	(0.09 to 0.13)	0.16	(0.11 to 0.21)
Ages_4	0.15	(-0.21 to 0.52)	0.29	(-0.13 to 0.71)	0.29	(0.25 to 0.33)	0.45	(0.31 to 0.59)
Birthyr_1	-0.01	(-0.04 to 0.02)	0.00	(-0.03 to 0.02)	-0.05	(-0.06 to -0.04)	-0.08	(-0.11 to -0.05)
Birthyr_2	0.00	(-0.01 to 0.02)	0.02	(-0.01 to 0.05)	-0.04	(-0.05 to -0.02)	-0.02	(-0.04 to 0.01)
Birthyr_3	0.01	(-0.04 to 0.07)	0.12	(-0.08 to 0.31)	-0.15	(-0.21 to -0.09)	0.06	(-0.11 to 0.24)
Birthyr_4	0.06	(-0.12 to 0.25)	0.42	(-0.44 to 1.29)	-0.54	(-0.77 to -0.30)	0.55	(-0.19 to 1.28)

DBP								
Exam year	-0.73	(-0.89 to -0.58)	-1.97	(-2.40 to -1.53)	-1.04	(-1.55 to -0.53)	-3.28	(-4.38 to -2.18)
Ages_1	0.03	(0.02 to 0.04)	0.01	(-0.01 to 0.03)	0.02	(0.01 to 0.03)	-0.02	(-0.04 to 0.01)
Ages_2	0.03	(0.02 to 0.04)	0.00	(-0.01 to 0.01)	0.02	(0.02 to 0.03)	0.00	(-0.02 to 0.01)
Ages_3	0.05	(0.03 to 0.08)	0.00	(-0.03 to 0.02)	0.05	(0.05 to 0.06)	0.00	(-0.02 to 0.03)
Ages_4	0.14	(0.06 to 0.21)	-0.02	(-0.08 to 0.03)	0.14	(0.12 to 0.16)	0.01	(-0.05 to 0.07)
Birthyr_1	-0.04	(-0.05 to -0.03)	-0.05	(-0.07 to -0.04)	-0.04	(-0.05 to -0.03)	-0.06	(-0.07 to -0.05)
Birthyr_2	-0.04	(-0.05 to -0.03)	-0.05	(-0.06 to -0.04)	-0.04	(-0.05 to -0.03)	-0.04	(-0.06 to -0.03)
Birthyr_3	-0.19	(-0.26 to -0.12)	-0.24	(-0.36 to -0.12)	-0.18	(-0.23 to -0.12)	-0.18	(-0.26 to -0.10)
Birthyr_4	-0.74	(-1.09 to -0.38)	-0.92	(-1.64 to -0.20)	-0.66	(-0.89 to -0.43)	-0.62	(-0.95 to -0.28)

BMI								
Exam year	0.36	(0.17 to 0.55)	0.98	(0.85 to 1.10)	-0.30	(-0.40 to -0.20)	-1.32	(-1.60 to -1.03)
Ages_1	0.01	(0.01 to 0.02)	0.02	(0.02 to 0.03)	0.01	(0.01 to 0.01)	0.01	(0.01 to 0.01)
Ages_2	0.01	(0.00 to 0.01)	0.01	(0.00 to 0.01)	0.01	(0.01 to 0.01)	0.01	(0.01 to 0.01)
Ages_3	0.01	(0.01 to 0.02)	0.00	(-0.01 to 0.01)	0.02	(0.02 to 0.02)	0.01	(0.00 to 0.01)
Ages_4	0.03	(0.01 to 0.05)	-0.01	(-0.04 to 0.01)	0.05	(0.04 to 0.05)	0.00	(-0.01 to 0.01)
Birthyr_1	0.00	(0.00 to 0.00)	0.00	(0.00 to 0.01)	-0.01	(-0.01 to -0.01)	-0.01	(-0.01 to -0.01)
Birthyr_2	0.00	(-0.01 to 0.00)	0.00	(0.00 to 0.00)	-0.02	(-0.02 to -0.02)	-0.03	(-0.03 to -0.02)
Birthyr_3	-0.02	(-0.04 to -0.01)	-0.01	(-0.05 to 0.02)	-0.12	(-0.13 to -0.10)	-0.07	(-0.09 to -0.04)
Birthyr_4	-0.10	(-0.18 to -0.02)	-0.11	(-0.31 to 0.10)	-0.47	(-0.61 to -0.33)	0.49	(-0.13 to 1.11)

TG								
Exam year	-1.27	(-2.76 to 0.23)	-0.05	(-2.23 to 2.13)	-2.32	(-3.32 to -1.31)	-7.62	(-9.39 to -5.85)
Ages_1	0.24	(-0.03 to 0.52)	0.28	(0.23 to 0.32)	0.13	(0.10 to 0.16)	0.06	(0.01 to 0.10)
Ages_2	0.13	(-0.16 to 0.42)	-0.13	(-0.16 to -0.09)	0.14	(0.12 to 0.16)	0.08	(0.05 to 0.12)
Ages_3	0.20	(-0.38 to 0.78)	-0.51	(-0.64 to -0.38)	0.30	(0.26 to 0.34)	0.19	(0.11 to 0.28)
Ages_4	0.36	(-1.00 to 1.72)	-1.83	(-2.27 to -1.40)	0.78	(0.69 to 0.87)	0.51	(0.29 to 0.74)
Birthyr_1	-0.23	(-0.44 to -0.03)	-0.23	(-0.26 to -0.20)	-0.17	(-0.20 to -0.13)	-0.22	(-0.26 to -0.19)
Birthyr_2	-0.29	(-0.44 to -0.13)	-0.44	(-0.49 to -0.39)	-0.15	(-0.19 to -0.11)	-0.18	(-0.24 to -0.12)
Birthyr_3	-1.53	(-2.21 to -0.84)	-2.52	(-3.19 to -1.84)	-0.69	(-0.90 to -0.48)	-0.73	(-1.08 to -0.37)
Birthyr_4	-6.10	(-8.48 to -3.72)	-10.16	(-13.80 to -6.53)	-2.59	(-3.45 to -1.72)	-2.41	(-3.93 to -0.88)

HDL								
Exam year	1.79	(0.59 to 2.99)	3.16	(1.47 to 4.84)	8.02	(2.95 to 13.09)	12.06	(10.39 to 13.74)
Ages_1	0.00	(-0.03 to 0.02)	0.04	(0.00 to 0.07)	0.05	(0.02 to 0.08)	0.00	(-0.02 to 0.02)
Ages_2	0.00	(-0.01 to 0.02)	0.04	(0.03 to 0.06)	0.05	(0.02 to 0.07)	0.00	(-0.02 to 0.02)
Ages_3	0.01	(-0.02 to 0.04)	0.09	(0.06 to 0.13)	0.10	(0.05 to 0.15)	0.00	(-0.05 to 0.06)
Ages_4	0.03	(-0.04 to 0.09)	0.24	(0.16 to 0.32)	0.25	(0.12 to 0.37)	0.00	(-0.18 to 0.18)
Birthyr_1	0.05	(0.03 to 0.06)	0.04	(0.03 to 0.06)	-0.05	(-0.08 to -0.02)	0.00	(-0.02 to 0.02)
Birthyr_2	0.05	(0.03 to 0.07)	0.05	(0.04 to 0.07)	-0.09	(-0.17 to -0.01)	0.00	(-0.07 to 0.08)
Birthyr_3	0.29	(0.18 to 0.40)	0.31	(0.23 to 0.39)	-0.80	(-1.42 to -0.18)	-0.07	(-0.64 to 0.50)
Birthyr_4	1.17	(0.73 to 1.61)	1.29	(0.97 to 1.62)	-5.80	(-10.19 to -1.42)	-2.31	(-7.51 to 2.89)

LDL								
Exam year	1.62	(-5.73 to 8.96)	10.45	(-27.47 to 48.38)	0.15	(-4.56 to 4.86)	-0.12	(-21.55 to 21.30)
Ages_1	0.13	(-0.13 to 0.39)	0.29	(-0.45 to 1.04)	0.11	(0.08 to 0.14)	0.12	(0.02 to 0.22)
Ages_2	0.10	(-0.04 to 0.24)	0.08	(-0.02 to 0.18)	0.12	(0.09 to 0.15)	0.13	(0.06 to 0.20)
Ages_3	0.19	(-0.05 to 0.43)	0.05	(-0.15 to 0.25)	0.26	(0.21 to 0.31)	0.28	(0.18 to 0.38)
Ages_4	0.45	(-0.02 to 0.91)	-0.16	(-1.49 to 1.16)	0.67	(0.56 to 0.78)	0.73	(0.54 to 0.91)
Birthyr_1	-0.07	(-0.12 to -0.02)	0.00	(-0.27 to 0.27)	-0.11	(-0.14 to -0.08)	-0.12	(-0.22 to -0.02)
Birthyr_2	-0.08	(-0.17 to 0.02)	-0.08	(-0.18 to 0.02)	-0.18	(-0.22 to -0.13)	-0.16	(-0.27 to -0.05)
Birthyr_3	-0.40	(-0.99 to 0.20)	-0.57	(-1.71 to 0.57)	-1.22	(-2.02 to -0.42)	-0.83	(-4.83 to 3.16)
Birthyr_4	-1.61	(-4.36 to 1.14)	-2.63	(-9.70 to 4.43)	-6.38	(-15.05 to 2.28)	-2.57	(-47.86 to 42.72)

UA								
Exam year	-0.04	(-0.08 to 0.00)	-0.16	(-0.24 to -0.08)	-0.17	(-0.27 to -0.06)	-0.22	(-0.37 to -0.07)
Ages_1	0.00	(0.00 to 0.00)	0.00	(-0.01 to 0.00)	0.00	(0.00 to 0.00)	0.00	(0.00 to 0.00)
Ages_2	0.00	(0.00 to 0.00)	0.00	(0.00 to 0.00)	0.00	(0.00 to 0.00)	0.00	(0.00 to 0.00)
Ages_3	-0.01	(-0.01 to 0.00)	-0.01	(-0.01 to 0.00)	0.01	(0.01 to 0.01)	0.01	(0.01 to 0.01)
Ages_4	-0.01	(-0.02 to -0.01)	-0.02	(-0.02 to -0.01)	0.03	(0.02 to 0.03)	0.03	(0.02 to 0.03)
Birthyr_1	0.00	(0.00 to 0.00)	0.00	(0.00 to 0.00)	0.00	(0.00 to 0.00)	0.00	(0.00 to 0.00)
Birthyr_2	0.00	(0.00 to 0.00)	0.00	(0.00 to 0.00)	0.01	(0.00 to 0.01)	0.01	(0.01 to 0.01)
Birthyr_3	0.01	(0.00 to 0.01)	0.00	(-0.01 to 0.01)	0.06	(0.02 to 0.10)	0.06	(0.01 to 0.10)
Birthyr_4	0.02	(0.00 to 0.03)	0.00	(-0.03 to 0.03)	0.36	(0.14 to 0.58)	0.29	(-0.20 to 0.79)

Linear PLSR showed that men examined at 2006 had nearly one-unit higher BMI, whilst women had nearly one-unit lower BMI, and these results were consistent with those from nonlinear PLSR. No substantive association between BMI and the year of birth or age at examination was found in either men or women.

In both linear and nonlinear models, no substantive association was found between SBP and any of the three covariates for men. In both linear and nonlinear models, there were small positive trends between SBP and age at examination in women. There was a decrease in DBP by 2 to 3 mmHg for men in 2006 and a similar decrease for women. Linear and nonlinear PLSR revealed small negative relationships between DBP and the year of birth in both men and women with a further decline around 1970.

Linear PLSR showed that the mean FPG in men examined at 2006 was about 5 mg/dl lower (1-component model: -4.2, 95% confidence interval [CI]: -5.86 to -2.55; 2-component model: -5.84, 95%CI: -7.72 to -3.95), whilst there was a small increase in FPG in women. These results were generally consistent with those from the nonlinear PLSR. Linear PLSR showed a small negative association between FPG and the year of birth for men and women. In the nonlinear PLSR, there was a negative trend in FPG and a fall for men born after 1970. A similar negative trend was observed in women with a smaller fall born at 1970. In both linear and nonlinear models, there were positive trends between FPG and age at examination.

Linear PLSR showed a decrease in TG for women (2-component model: -9.58, 95%CI: -12.71 to -6.45). Linear PLSR revealed small negative relationships between TG and the year of birth in both men and women. Nonlinear PLSR also yielded negative trends, though with a further decline around 1970. No substantive association was found between TG and age at examination.

In both linear and nonlinear models, there were increases in HDL in both men and women between 2006 and 1996. PLSR showed a small positive association between HDL and the year of birth in men with a sharp rise around 1970. The association between HDL and the year of birth for women was less clear, as linear and nonlinear models suggested different trends. Both linear and nonlinear PLSR models suggested no strong association between HDL and age.

The adjusted difference in mean LDL between 2006 and 1996 for men and women had very large confidence intervals in both linear and nonlinear PLSR, yielding inconclusive interpretation. Linear PLSR revealed very small negative relationships between LDL and the year of birth in both men and women. Nonlinear PLSR also yielded negative trends, though with a further decline around 1970 (Figure 3). Linear and nonlinear PLSR suggested small positive associations between LDL and age at examination.

The adjusted UA was lower in 2006 for men and women in both linear and nonlinear PLSR. Linear PLSR showed no relationships between LDL and the year of birth in both men and women. Nonlinear PLSR, however, suggested a sudden increase in UA in women around 1970 (Figure 3). Linear and nonlinear PLSR suggested a weak negative association between UA and age at examination in men.

## Discussion

Our analyses suggest that whilst there was a small increase in BMI from 1996 to 2006 amongst men, there was a small decrease amongst women. One recent study also found an increase in the prevalence of obesity in Taiwanese men but not women between 1993/6 and 2005 [16]. This sex difference may be down to women becoming taller whilst their weight remains similar, though intriguingly the other seven metabolic syndrome components either showed little change or became slightly better for both men and women. It may be hypothesized that social pressures to be maintain slimness could be different in men and women and that this is driving the differential effect of gender. While men became taller and larger in 2006, women became taller but with similar weights. The mean DBP was lower in 2006 than in 1996 in this population [22], but the present study also observed lower TG, LDL, UA and higher HDL in all age groups. There was a small increase in FPG for men in 2006 compared to 1996, and the difference was larger in older age groups than younger ones; however, no difference in FPG was observed for women. The increased BMI in men is consistent with the increased prevalence of obesity suggested by other studies, but this did not necessarily reflect an increased risk for metabolic syndrome in this study.

Several authors have argued that obesity is not only a serious public health issue for developed countries but also for developing countries that are undergoing or have recently undergone economic and social transformation, when people gradually adopt western lifestyles and diets. Our hypothesis was that changes in lifestyles and the environment, arising due to economic development, may have counteractive impacts on population health; this idea is new and to the best of our knowledge has not been investigated before. While attention has been focused on the potentially harmful impact of the changing environment in adulthood on health in later life, less is known about the potentially protective effects of an improved early environment. For people who experience a nutritional and lifestyle revolution due to economic and social transformation in early and middle adulthood, the mismatch in the early and later environments may increase their risks of chronic diseases in later life probably mediated by obesity [4-6]. However, such a mismatch becomes small for later generations who grow-up in the later stage of economic and social transformation, and moreover, the improved nutrition and living standards for mothers and babies may give these later generations improved health compared to those before them.

Traditionally, to unravel the differential effects of the changing environment on successive generations requires a complex age-period-cohort analysis [31], because three factors need to be considered simultaneously. The first is the period effect, i.e. the "current" or later-life environment, and this is usually when health data are collected. The second is the cohort effect, i.e. the early environment for which the year of birth is usually a proxy. The third factor, the participants' age when the data are collected, also needs to be taken into account, because health changes with age. The long-standing problem, however, in this age-period-cohort analysis is that it has not been possible to separate the independent effects of the three factors by undertaking a standard regression analysis [31,32]. This is because mathematically cohort + age = period, and the usual regression analysis approach to estimating the "independent" effect of one variable by adjusting for the other two becomes impossible.

**Figure 4:**
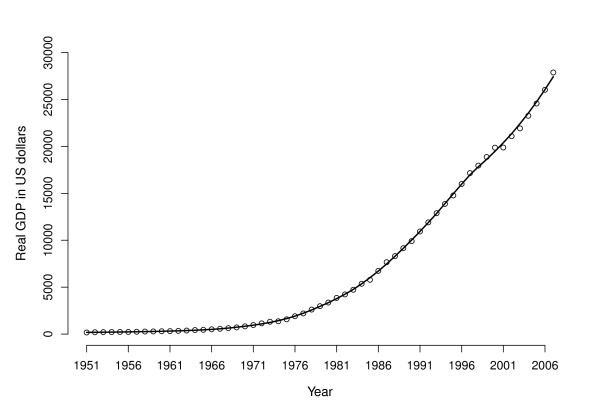
**Trends in the real gross domestic product (GDP) in Taiwan between year 1951 and 2007**.

To overcome this problem, we used PLSR to separate out the age-period-cohort effects. PLSR can estimate the individual effects of the three variables by partitioning the total effect according to the covariance structures amongst them and the outcome. As there is no reason to believe that these effects should be linear, we undertook both linear and nonlinear PLSR. We examine results from the one- and two-component models to check the robustness of the nonlinear PLSR and to avoid over-interpretation, even though PRESS indicated a preference for the one-component models. For linear PLSR, results from the one-component and the two-component models are quite consistent (Table 3). For nonlinear PLSR, most results were quite consistent except for TG and FPG for men. It should be noted that the trends plotted in Figure 4 were the average based on the point estimates, but we should not forget that these point estimates come with confidence intervals and, therefore, there is always uncertainty associated with these trends. This is why we present results from both the one-component and two-component models to examine the robustness of our findings. As it is not yet possible to plot confidence intervals for the trends, since both the one-component and two-component models show similar results, this provides some reassurance regarding the robustness of the one-component model; however, if results from the one-component and two-component models were inconsistent, we would need to be conservative in the interpretation given to the trends in either model. This also applies to the interpretation of linear PLSR, where consistency within one-component and two-component models should be examined against not only point estimates, but also confidence intervals. Where confidence intervals in general overlap, this indicates consistency in the findings.

Some of the results from PLSR are consistent with the simple summary statistics in Table 1, such as the association between age and some metabolic syndrome components. We found that DBP was lower for both men and women and SBP was lower for women in 2006. However, we also found that TG was lower and HDL higher in 2006. These results seem to suggest that the risk for metabolic syndrome might not increase, as expected, when the prevalence of obesity increases in the context of economic growth continuing in Taiwan. One explanation is that people who attended the health screening clinics were more affluent and therefore have a greater awareness of health-related issues, such as diet and physical activity. Consequently, the association between change in BMI and other components becomes less clear. If this were true, the focus of public health should therefore be around lifestyles and diet changes rather than BMI or weight changes.

The most intriguing finding in this study is the association between the year of birth and metabolic syndrome components. Whilst there were general trends in the many associations observed [usually better outcomes amongst people born more recently], nonlinear PLSR consistently showed a sudden shift in these associations around the period of 1970 (Figure 3). One explanation is that being mathematically related, people born earlier were also older at the time of examination, so they tended to have less favorable metabolic syndrome profile. Whilst this might be plausible as "residual" confounding of age cannot be completely ruled out in PLSR or in any observational studies, it does not explain why in HDL, for women, age and birth year had the same positive trend and why there is the sudden decline/increase around 1970. The effect of age tended to become stronger after age 40, corresponding to people born before 1956 or 1966 who undertook health checks in 1996 or 2006, respectively. Therefore, any residual confounding of age would suggest a sudden increase/decline in the relation with year of birth taking place during this period, not around the period of 1970 or thereafter.

An alternative explanation is the protective effect of improved early environment. Figure 4 shows the trend in the mean real gross domestic product (GDP) in Taiwan between 1951 and 2006 (from the website at the University of Pennsylvania: http://pwt.econ.upenn.edu, accessed on 13^th ^June 2010). There is a strikingly similar pattern between the trend in GDP growth and in many of the relationships of metabolic syndrome components with the year of birth. If the year of birth is taken as a proxy variable for the nutritional and social environment in early life, our results suggest that the impact of economic and social transformation on public health may not be always deleterious in countries with rapidly growing economy. Such transformations may have two possible effects working counteractively, and public policy should aim to enhance or at least embrace the beneficial impacts, whilst working against the negative impacts.

The similarity in the trends was observed in only some metabolic syndrome components, which may suggest a differential effect of early environment on metabolic syndrome by sex and differential effects on the components. It is likely that there may be a threshold effect, i.e. when economic growth and the accompanying social transformation attain a certain level, the effect on population health becomes notable. While the economy started growing since 1950s, Taiwanese economy only really took off in the early 1970s. Increased wealth improved living standards, such as housing and nutrition, and it also provided more investment in education and medical care. People started to think how to live not only longer but better, and this is why private health screening services became popular in Taiwan since the 1990s.

There are also some limitations in this study: first, participants with previous medical histories or medications were excluded from the analyses, and this may give rise to selection bias [33]. Second, while PLSR can separate the effects of age, period and cohort, there may be interactions among them that have not been evaluated here. To test for these potential interactions would substantially increase the complexity of our statistical models and is beyond the scope of this study. Third, although the cubic splines method provides an elegant way of examining nonlinear relationships, there are more sophisticated ways of specifying nonlinear associations in PLSR, such as penalized PLSR [34], which is more advanced mathematically but less intuitive in the interpretation of results.

There has been a debate about whether the impact of an obesity epidemic on diseases, such as type 2 diabetes and cardiovascular diseases, has been exaggerated [35-37]. For instance, one study on secular trends in cardiovascular disease risk factors in US adults found that the prevalence of obesity has increased in recent decades; however, whilst the prevalence of diabetes has also increased, cardiovascular risk factors, such as high cholesterol and high blood pressure level, have declined considerably [38]. Another study showed a decreased magnitude of association between blood pressure and BMI in a survey undertaken in 1989 and 2005 in the Seychelles, a rapidly developing country in Africa [39], whilst the average BMI in 2006 was greater than that in 1989. In both economically developed and developing countries, there have been changes in deleterious and beneficial factors, such as physical activities, smoking, consumption of fruit and vegetables, dietary patterns and education [38]. There may be differential changes in those factors across different populations. For example, whilst people living in the cities may on average have lower physical activity, the more affluent can afford access to facilities such as sports gymnasiums, whilst the poor do not. People with higher education are likely to steer clear of processed food and consume more fresh fruits and vegetables. The risk for diabetes and cardiovascular diseases is a result of interactions amongst many factors, and may not be captured well by a single obesity index, such as adult BMI. To inform effective policy for implementing public health programs, a comprehensive, life course approach is required to identify variables working at different (population, personal and genetic) levels and their impact on health at different phases throughout the life course.

## Conclusions

Our age-period-cohort analysis of a Taiwanese cohort suggests that changing environment might have two possible effects working counteractively in a country with rapid economic and social changes; the risk for metabolic syndrome does not necessarily increase with the prevalence of obesity. Public policy should therefore aim to enhance or at least embrace the beneficial impacts, whilst working against the negative impacts.

## Abbreviations

(BMI): body mass index; (SBP): systolic blood pressure; (DBP): diastolic blood pressure; (FPG): fasting plasma glucose; (TG): triglycerides; (HDL): high-density lipoprotein; (LDL): Low-density lipoproteins; (UA): uric acid

## Competing interests

The authors declare that they have no competing interests.

## Authors' contributions

YKT conceived the ideas, took the initiative to acquire the data, carried out the statistical analyses and drafted the manuscript. KLC contributed to the acquisition of the data, generation of research hypotheses, interpretation of results and critical revisions to the manuscript. VC contributed to the generation of research hypotheses, interpretation of results and critical revisions to the manuscript. MSG contributed to the acquisition of data, generation of research hypotheses, interpretation of results and critical revisions to the manuscript. All authors have approved the final content of this manuscript.

## Appendix

### Identification problem in the Age-Period-Cohort (APC) analysis

The main problem with the APC analysis is the intrinsic mathematical relation amongst *Age*, *Period *and *Cohort*. For instance, the relationship between systolic blood pressure (*SBP*) and the three variables, *Age *(chronological age at examination), *Period *(year at examination) and *Cohort *(year of birth) in an ordinary least squares (OLS) regression are written as:(A-1)

where *b*_0 _is the intercept, *b*_1_, *b*_2 _and *b*_3 _are the regression coefficients for *Age*, *Period *and *Cohort*, respectively, and *e *is the residual error term. To simplify our discussion, we assume that all the four variables are centered, i.e. their means have been subtracted from initial individual values for each variable. Therefore, we can exclude the intercept in equation (A-1) from the model. In matrix notation, equation (A-1) can be expressed as:(A-2)

where **y **is a vector for *body weight*, and *X *is the design matrix for *Age*, *Period*, and *Cohort*, **b **is a vector for *b*_1_, *b*_2 _and *b*_3_, and **e **is a vector for the residuals. The estimation for **b **is to solve the following equation [40-43]:(A-3)

where *X*^*T *^is the transposed matrix of *X*, and (*X*^*T*^*X*)^-1 ^is the inverse of *X*^*T*^*X*.

Since *Age *+ *Cohort *= *Period*, statistical software packages cannot proceed with computation unless at least one of the three covariates is removed from the model [31,32]. This is because the product matrix *X*^*T*^*X *is not full-rank and consequently (*X*^*T*^*X*)^-1 ^does not exist. However, the problem is not that there is no solution to **b**, but that there are "too many" solutions, i.e. there is no unique solution to *b*_1_, *b*_2 _and *b*_3_, unless some constraints are imposed on the estimation of **b **[31,32]. From a mathematical viewpoint, this is because whilst (*X*^*T*^*X*)^-1 ^does not exist, there are indefinite numbers of generalized inverse matrices for *X*^*T*^*X*, (*X*^*T*^*X*)^-^. When *X*^*T*^*X *is full-rank, it can be shown that (*X*^*T*^*X*)^- ^is unique and is equivalent to (*X*^*T*^*X*)^-1 ^[40,41].

Amongst the indefinite number of generalized inverse matrices, one special and unique generalized inverse matrix of *X*^*T*^*X*, known as the Moore-Penrose inverse, (*X*^*T*^*X*)^+^, has been widely used in statistics to resolve the identification problem [40,41]. The Moore-Penrose inverse is closely related to a mathematical technique known as singular value decomposition (SVD) in matrix algebra [40-43]. It is well known that results from the use of the Moore-Penrose inverse is equivalent to those from principal component regression (PCR) and partial least squares regression (PLSR) when the maximum number of components is retained [44-46].

For the APC analysis, obtaining any solution requires the imposition of a constraint in the estimation of **b **in equation (A-1), and whether or not the solution is meaningful depends upon the choice of constraint, i.e. the conditions imposed in estimation. This principle applies in general to all the "solutions" proposed in the literature on the APC analysis. In the next sections, we seek to explore the statistical conditions imposed by PLSR.

### Partial Least Squares Regression (PLSR) and perfect collinearity

In PCR, the extraction of components is independent of the outcome variable, i.e. the same components are extracted in the same order as new covariates irrespective of the outcome. From a data reduction point of view, this is not always desirable if the aim is to find a parsimonious model for predicting the outcome, because sometimes principal components with large variances may have low correlations with the outcome [47]. This potential weakness is amended in PLSR, as the extraction of components in PLSR aims to maximize the covariance with the outcome *y*:(A-4)

under the same constraints for PCR that |**w**_*i*_**| = 1 **and **w**_*i *_⊥ **w**_*j *_(*i *≠ *j*) [44-46].

The first partial least squares component therefore has the largest covariance with the outcome and the second component has the second largest covariance, etc. PLSR is also a data-dimension reduction method, and usually only the first few components are retained as new covariates, which therefore explain most of the variance in the outcome that can be explained by the original covariates. When there is no perfect collinearity in *X *and *n *>>*p*, results from OLS, PCR and PLSR are equivalent if *p *components are retained as covariates; otherwise, they will yield different results. If there is perfect collinearity in *X*, or if *n *<*p*, results from PCR and PLSR are equivalent when *r *components are retained, where *r *is the column rank of *X *[44-46]; otherwise, results are different.

PLSR was first developed as a set of algorithms to extract components in an iterative process, but it was then shown that PLSR is related to a series of SVD of *X*^*T*^**y **[45,48]. Taking the association between *SBP *and the *Age*, *Period*, and *Cohort *in men as an example, the matrix *X*^*T*^**y **(where all four variables are centred) is:

Astute readers may notice that 35105.27 + (- 62852.03) = (-27746.77), i.e. the sum of the first and third elements is equal to the second, which corresponds to the simple mathematical relation *Age *+ *Cohort *= *Period*. The proof for this observation is simple: let us call *Age x*_1_, *Period x*_2_, *Cohort x*_3 _and *SBP y*, i.e. *x*_1 _+ *x*_3 _= *x*_2_. After subtracting the mean from each variable, we find:(A-5)

where ,  and  are the means of *x*_1_, *x*_2_, and *x*_3, _respectively. Multiplying both sides of equation (A-5) by  (where  is the mean of *y*), we obtain the equality observed in *X*^*T*^**y**. We now undertake singular value decomposition (SVD) for *X*^*T*^**y**:

The singular value 77153.36 is the squared root of the sum of squares of each element in *X*^T^**y**: (35105.27)^2 ^+ (-27746.77)^2 ^+ (-62853.03)^2 ^= (77153.36)^2^. 77153.36 divided by 20707 (the sample size minus 1) is 3.726, and its square 13.88 is the sum of the variance of the three projected vectors of *X *on **y **(if we undertake the singular value analysis for *X*^*T*^**yy**^**T**^*X *instead, the first singular value will be 13.88 and the other singular values are zero)[45,48]. Image we project *x*_1_, *x*_2_, and *x*_3 _on **y**, and obtain three orthogonally projected vectors **ŷ**_1_, **ŷ**_2_, and **ŷ**_3_, respectively. These three vectors will be either in the same or the opposite direction, and **ŷ**_1_+ **ŷ**_3_= **ŷ**_2_, as they only span 1 dimension. The total variance of the three vectors is then 13.88, i.e. . Note that the left singular vector [0.455, -0.360, -0.815]^T ^contains the weights for the first PLS component (**t**_1_), i.e.:

When *SBP *is regressed on **t**_1_, the regression coefficient is 0.026, and the PLSR model with 1 component can be written as:

Therefore, the PLSR coefficients *b*_1_, *b*_2 _and *b*_3_, also satisfy the simple mathematical relation *b*_1 _+ *b*_3 _= *b*_2 _(i.e. 0.012 + (-0.021) = -0.009). Although partial least squares algorithms as described in the literature [44-46,48] do not make explicit constraints on the estimation of regression coefficients, the mathematical relation amongst the three covariates give rise to an implicit constraint. In fact, such a constraint in the estimation of **b **has been proposed in the literature based on a geometric idea [49].

### Scaling of covariates

In PLSR and PCR, covariates are usually scaled to have unit variance, because PLSR and PCR penalizes covariates with small variances. For instance, *Period *(the year of examination) in our study has the smallest variance because it has only a range of 10 years. In order not to penalize *Period*, we scaled the three covariates, and this is equivalent to giving differential weighting in the constraint during the estimation process, i.e. the simple constraint that *b*_1 _+ *b*_3 _= *b*_2 _becomes:(A-6)

where *b*_1c_, *b*_2c _and *b*_3c_, are PLSR coefficients when covariates are scaled in the extraction of partial least square components. Simple algebra shows that the differential weighting is:(A-7)

where *s*_1_, *s*_2 _and *s*_3 _are the standard deviations of *Age*, *Period *and *Cohort*, respectively. For example, in Table 3, PLSR coefficients for *Age*, *Period *and *Cohort *are 0.01, -0.49 and -0.02. It can be verified that:

The small inconsistency is due to rounding errors. Thus, PLSR makes an implicit constraint to achieve identification for linear models with perfectly collinear variables and this knowledge is essential for the interpretation of the results from analyses using these methods.

In summary, PLSR partitions the total effect of *Age*, *Period *and *Cohort *on *SBP *according to the relationships between *SBP *and the three covariates and the relationships amongst the perfectly collinear covariates by imposing an implicit constraint on the relationship amongst the regression coefficients. We call the constraint implicit because this constraint is not intentionally imposed by the algorithms; instead, the constraint arises from the intrinsic mathematical relationship amongst the perfectly collinear covariates. As explained at the start of the appendix, any solution to the identification problem must impose some constraint on the estimation of regression coefficients. Our theoretical exploration shows that the constraint imposed for APC analyses by PLSR seems to be a justifiable one. For instance, since *Age *+ *Cohort *= *Period*, it seems quite reasonable to assume that the sum of the effects of *Age *and *Cohort *should be equal to the effect of *Period*. When the variances of the three variables are not equal, we give differential weighting to the constraint.

For the nonlinear analyses in this study, where polynomial spline terms were used to model the nonlinear relationships, the partial least squares regression coefficients for those polynomial terms were not affected by the collinearity amongst the linear functional terms. For example, when one of *Age*, *Period *and *Cohort *is removed from the model, this will not change the regression coefficients for the polynomial terms. This is because the identification problem is local to the linear terms, and this does not affect the estimation of polynomial terms [40,41].

## Pre-publication history

The pre-publication history for this paper can be accessed here:

http://www.biomedcentral.com/1471-2288/11/82/prepub

## Supplementary Material

Additional file 1 **Complete Tables for Body size, components of the metabolic syndrome, and aspects of lifestyle in 1996 and 2006 for men (Table 1) and women (Table 2)**. Click here for file
