# Supplementary material for: Unravelling the effects of age, period and cohort on metabolic syndrome components in a Taiwanese population using partial least squares regression
Source: BMC Med Res Methodol. 2011 May 27;11:82. doi: 10.1186/1471-2288-11-82 (PMC3117818; doi:10.1186/1471-2288-11-82)
Supplement: Additional file 1 — Complete Tables for Body size, components of the metabolic syndrome, and aspects of lifestyle in 1996 and 2006 for men (Table 1) and women (Table 2). [file 1471-2288-11-82-S1.DOC]

# Supplement material: complete version of Tables 1 and 2

**Table 1**: Body size, components of the metabolic syndrome, and aspects of lifestyle in 1996 and 2006 for men.

|  |  | Total |  |  |  | 20-29 |  |  |  | 30-39 |  |  |  | 40-49 |  |  |  | 50-59 |  |
| --- | --- | --- | --- | --- | --- | --- | --- | --- | --- | --- | --- | --- | --- | --- | --- | --- | --- | --- | --- |
|  | N | Mean | SD |  | N | Mean | SD |  | N | Mean | SD |  | N | Mean | SD |  | N | Mean | SD |
| Year 1996 |  |  |  |  |  |  |  |  |  |  |  |  |  |  |  |  |  |  |  |
| Body weight (kg) | 6520 | 68.00 | 10.38 |  | 1940 | 66.70 | 10.97 |  | 2762 | 68.82 | 10.63 |  | 1192 | 69.10 | 9.23 |  | 626 | 66.35 | 8.69 |
| Body height (cm) | 6520 | 169.58 | 6.00 |  | 1940 | 171.04 | 5.97 |  | 2762 | 169.96 | 5.83 |  | 1192 | 168.42 | 5.60 |  | 626 | 165.56 | 5.42 |
| BMI (kg/m^2^) | 6520 | 23.63 | 3.22 |  | 1940 | 22.77 | 3.33 |  | 2762 | 23.80 | 3.25 |  | 1192 | 24.34 | 2.88 |  | 626 | 24.19 | 2.77 |
| FPG (mg/dl) | 6520 | 96.87 | 14.51 |  | 1940 | 94.13 | 11.68 |  | 2762 | 96.26 | 11.10 |  | 1192 | 99.76 | 17.30 |  | 626 | 102.52 | 24.21 |
| SBP (mmHg) | 6520 | 117.99 | 14.66 |  | 1940 | 117.87 | 13.83 |  | 2762 | 116.80 | 13.67 |  | 1192 | 118.08 | 15.23 |  | 626 | 123.46 | 18.55 |
| DBP (mmHg) | 6520 | 73.07 | 9.83 |  | 1940 | 71.06 | 9.29 |  | 2762 | 72.84 | 9.51 |  | 1192 | 75.26 | 10.32 |  | 626 | 76.11 | 10.36 |
| TG (mg/dl) | 6520 | 131.81 | 104.38 |  | 1940 | 106.65 | 79.54 |  | 2762 | 140.33 | 110.73 |  | 1192 | 148.19 | 111.83 |  | 626 | 140.95 | 114.63 |
| HDL (mg/dl) | 6519 | 41.27 | 12.18 |  | 1940 | 40.93 | 11.19 |  | 2762 | 41.04 | 12.49 |  | 1192 | 41.50 | 12.34 |  | 626 | 42.88 | 13.29 |
| LDL (mg/dl) | 6401 | 125.52 | 31.31 |  | 1924 | 116.94 | 28.45 |  | 2695 | 125.79 | 30.62 |  | 1165 | 134.24 | 32.52 |  | 617 | 134.60 | 33.18 |
| UA (mg/dl) | 6520 | 6.88 | 1.36 |  | 1940 | 6.93 | 1.36 |  | 2762 | 6.95 | 1.37 |  | 1192 | 6.81 | 1.32 |  | 626 | 6.60 | 1.37 |
|  | N | % |  |  | N | % |  |  | N | % |  |  | N | % |  |  | N | % |  |
| Education |  |  |  |  |  |  |  |  |  |  |  |  |  |  |  |  |  |  |  |
| ≤ 9 year | 998 | 15.31 |  |  | 91 | 4.69 |  |  | 260 | 9.41 |  |  | 254 | 21.31 |  |  | 393 | 62.78 |  |
| 10-15 years | 3,817 | 58.54 |  |  | 1,378 | 71.03 |  |  | 1,692 | 61.26 |  |  | 578 | 48.49 |  |  | 169 | 27 |  |
| ≥ 15 years | 1,705 | 26.15 |  |  | 471 | 24.28 |  |  | 810 | 29.33 |  |  | 360 | 30.2 |  |  | 64 | 10.22 |  |
| Smoking |  |  |  |  |  |  |  |  |  |  |  |  |  |  |  |  |  |  |  |
| NA | 333 | 5.11 |  |  | 92 | 4.74 |  |  | 139 | 5.03 |  |  | 61 | 5.12 |  |  | 41 | 6.55 |  |
| Never | 2,683 | 41.15 |  |  | 900 | 46.39 |  |  | 1,051 | 38.05 |  |  | 507 | 42.53 |  |  | 225 | 35.94 |  |
| Previously | 566 | 8.68 |  |  | 99 | 5.1 |  |  | 256 | 9.27 |  |  | 138 | 11.58 |  |  | 73 | 11.66 |  |
| Occasionally | 365 | 5.6 |  |  | 117 | 6.03 |  |  | 159 | 5.76 |  |  | 60 | 5.03 |  |  | 29 | 4.63 |  |
| Frequently | 370 | 5.67 |  |  | 105 | 5.41 |  |  | 171 | 6.19 |  |  | 52 | 4.36 |  |  | 42 | 6.71 |  |
| Every day | 2,203 | 33.79 |  |  | 627 | 32.32 |  |  | 986 | 35.7 |  |  | 374 | 31.38 |  |  | 216 | 34.5 |  |
| Alcohol Drinking* |  |  |  |  |  |  |  |  |  |  |  |  |  |  |  |  |  |  |  |
| NA | 236 | 3.62 |  |  | 70 | 3.61 |  |  | 91 | 3.29 |  |  | 49 | 4.11 |  |  | 26 | 4.15 |  |
| Never | 2,713 | 41.61 |  |  | 935 | 48.2 |  |  | 1,108 | 40.12 |  |  | 429 | 35.99 |  |  | 241 | 38.5 |  |
| Previously | 123 | 1.89 |  |  | 31 | 1.6 |  |  | 52 | 1.88 |  |  | 23 | 1.93 |  |  | 17 | 2.72 |  |
| Occasionally | 2,542 | 38.99 |  |  | 737 | 37.99 |  |  | 1,109 | 40.15 |  |  | 489 | 41.02 |  |  | 207 | 33.07 |  |
| Frequently | 755 | 11.58 |  |  | 153 | 7.89 |  |  | 349 | 12.64 |  |  | 164 | 13.76 |  |  | 89 | 14.22 |  |
| Every day | 151 | 2.32 |  |  | 14 | 0.72 |  |  | 53 | 1.92 |  |  | 38 | 3.19 |  |  | 46 | 7.35 |  |
| Use of betel nuts |  |  |  |  |  |  |  |  |  |  |  |  |  |  |  |  |  |  |  |
| NA | 178 | 2.73 |  |  | 27 | 1.39 |  |  | 80 | 2.9 |  |  | 44 | 3.69 |  |  | 27 | 4.31 |  |
| Never | 4,615 | 70.78 |  |  | 1,356 | 69.9 |  |  | 1,902 | 68.86 |  |  | 893 | 74.92 |  |  | 464 | 74.12 |  |
| Occasionally | 1,372 | 21.04 |  |  | 465 | 23.97 |  |  | 621 | 22.48 |  |  | 194 | 16.28 |  |  | 92 | 14.7 |  |
| Frequently | 202 | 3.1 |  |  | 56 | 2.89 |  |  | 96 | 3.48 |  |  | 31 | 2.6 |  |  | 19 | 3.04 |  |
| Every day | 153 | 2.35 |  |  | 36 | 1.86 |  |  | 63 | 2.28 |  |  | 30 | 2.52 |  |  | 24 | 3.83 |  |
|  |  |  |  |  |  |  |  |  |  |  |  |  |  |  |  |  |  |  |  |
|  | N | Mean | SD |  | N | Mean | SD |  | N | Mean | SD |  | N | Mean | SD |  | N | Mean | SD |
| Year 2006 |  |  |  |  |  |  |  |  |  |  |  |  |  |  |  |  |  |  |  |
| Body weight (kg) | 14261 | 70.76 | 10.79 |  | 3109 | 70.54 | 11.89 |  | 6292 | 71.55 | 10.90 |  | 3538 | 70.37 | 9.82 |  | 1322 | 68.53 | 9.53 |
| Body height (cm) | 14261 | 171.56 | 6.08 |  | 3109 | 173.02 | 5.88 |  | 6292 | 172.21 | 5.88 |  | 3538 | 170.52 | 5.90 |  | 1322 | 167.76 | 5.93 |
| BMI (kg/m^2^) | 14261 | 24.01 | 3.24 |  | 3109 | 23.54 | 3.65 |  | 6292 | 24.10 | 3.25 |  | 3538 | 24.18 | 2.94 |  | 1322 | 24.31 | 2.83 |
| FPG (mg/dl) | 14258 | 98.55 | 14.31 |  | 3109 | 94.80 | 9.07 |  | 6289 | 97.58 | 12.18 |  | 3538 | 101.42 | 17.90 |  | 1322 | 104.25 | 18.97 |
| SBP (mmHg) | 14259 | 119.72 | 13.69 |  | 3109 | 120.10 | 12.61 |  | 6291 | 119.19 | 13.12 |  | 3537 | 119.22 | 14.40 |  | 1322 | 122.65 | 16.22 |
| DBP (mmHg) | 14259 | 71.38 | 9.95 |  | 3109 | 68.76 | 9.02 |  | 6291 | 71.02 | 9.42 |  | 3537 | 72.98 | 10.47 |  | 1322 | 74.98 | 11.18 |
| TG (mg/dl) | 14259 | 128.30 | 98.03 |  | 3109 | 97.39 | 61.81 |  | 6290 | 131.77 | 92.44 |  | 3538 | 146.24 | 124.72 |  | 1322 | 136.44 | 95.74 |
| HDL (mg/dl) | 13300 | 48.05 | 10.89 |  | 2918 | 49.69 | 10.70 |  | 5843 | 47.30 | 10.77 |  | 3269 | 47.60 | 10.96 |  | 1270 | 48.93 | 11.34 |
| LDL (mg/dl) | 13294 | 122.29 | 30.96 |  | 2917 | 112.86 | 28.70 |  | 5837 | 122.56 | 30.32 |  | 3269 | 127.75 | 31.80 |  | 1271 | 128.66 | 31.56 |
| UA (mg/dl) | 14250 | 6.56 | 1.23 |  | 3109 | 6.68 | 1.24 |  | 6288 | 6.62 | 1.23 |  | 3532 | 6.41 | 1.20 |  | 1321 | 6.35 | 1.21 |
|  | N | % |  |  | N | % |  |  | N | % |  |  | N | % |  |  | N | % |  |
| Education |  |  |  |  |  |  |  |  |  |  |  |  |  |  |  |  |  |  |  |
| ≤ 9 year | 853 | 5.98 |  |  | 87 | 2.8 |  |  | 185 | 2.94 |  |  | 263 | 7.43 |  |  | 318 | 24.05 |  |
| 10-15 years | 5,534 | 38.8 |  |  | 965 | 31.04 |  |  | 2,389 | 37.97 |  |  | 1,653 | 46.71 |  |  | 527 | 39.86 |  |
| ≥ 15 years | 7,875 | 55.22 |  |  | 2,057 | 66.16 |  |  | 3,718 | 59.09 |  |  | 1,623 | 45.86 |  |  | 477 | 36.08 |  |
| Smoking |  |  |  |  |  |  |  |  |  |  |  |  |  |  |  |  |  |  |  |
| NA | 269 | 1.89 |  |  | 33 | 1.06 |  |  | 92 | 1.46 |  |  | 95 | 2.68 |  |  | 49 | 3.71 |  |
| Never | 7,750 | 54.34 |  |  | 1,876 | 60.34 |  |  | 3,399 | 54.02 |  |  | 1,751 | 49.48 |  |  | 724 | 54.77 |  |
| Secondhand | 415 | 2.91 |  |  | 139 | 4.47 |  |  | 188 | 2.99 |  |  | 69 | 1.95 |  |  | 19 | 1.44 |  |
| Previously | 981 | 6.88 |  |  | 124 | 3.99 |  |  | 403 | 6.4 |  |  | 335 | 9.47 |  |  | 119 | 9 |  |
| Occasionally | 918 | 6.44 |  |  | 187 | 6.01 |  |  | 442 | 7.02 |  |  | 226 | 6.39 |  |  | 63 | 4.77 |  |
| Every day | 3,929 | 27.55 |  |  | 750 | 24.12 |  |  | 1,768 | 28.1 |  |  | 1,063 | 30.04 |  |  | 348 | 26.32 |  |
| Alcohol drinking* |  |  |  |  |  |  |  |  |  |  |  |  |  |  |  |  |  |  |  |
| NA | 363 | 2.55 |  |  | 49 | 1.58 |  |  | 125 | 1.99 |  |  | 118 | 3.33 |  |  | 71 | 5.37 |  |
| Never | 10,460 | 73.34 |  |  | 2,545 | 81.86 |  |  | 4,717 | 74.97 |  |  | 2,374 | 67.08 |  |  | 824 | 62.33 |  |
| Previously | 244 | 1.71 |  |  | 39 | 1.25 |  |  | 99 | 1.57 |  |  | 67 | 1.89 |  |  | 39 | 2.95 |  |
| 1-2 times/week | 2,247 | 15.76 |  |  | 375 | 12.06 |  |  | 980 | 15.58 |  |  | 659 | 18.62 |  |  | 233 | 17.62 |  |
| 3-4 times/week | 677 | 4.75 |  |  | 79 | 2.54 |  |  | 285 | 4.53 |  |  | 218 | 6.16 |  |  | 95 | 7.19 |  |
| Every day | 271 | 1.9 |  |  | 22 | 0.71 |  |  | 86 | 1.37 |  |  | 103 | 2.91 |  |  | 60 | 4.54 |  |
| Use of betel nuts |  |  |  |  |  |  |  |  |  |  |  |  |  |  |  |  |  |  |  |
| NA | 345 | 2.42 |  |  | 58 | 1.87 |  |  | 143 | 2.27 |  |  | 102 | 2.88 |  |  | 42 | 3.18 |  |
| Never | 12,415 | 87.05 |  |  | 2,759 | 88.74 |  |  | 5,455 | 86.7 |  |  | 3,064 | 86.58 |  |  | 1,137 | 86.01 |  |
| Previously | 931 | 6.53 |  |  | 180 | 5.79 |  |  | 429 | 6.82 |  |  | 232 | 6.56 |  |  | 90 | 6.81 |  |
| 1-3 times/week | 409 | 2.87 |  |  | 83 | 2.67 |  |  | 196 | 3.12 |  |  | 105 | 2.97 |  |  | 25 | 1.89 |  |
| 4-5 times/week | 63 | 0.44 |  |  | 12 | 0.39 |  |  | 29 | 0.46 |  |  | 12 | 0.34 |  |  | 10 | 0.76 |  |
| Every day | 99 | 0.69 |  |  | 17 | 0.55 |  |  | 40 | 0.64 |  |  | 24 | 0.68 |  |  | 18 | 1.36 |  |

*Questions about alcohol drinking were slightly modified after 1996.

**Table 2:** Body size, components of the metabolic syndrome, and aspects of lifestyle in 1996 and 2006 for women.

|  | | | | | | |  | | | | | Total | | | |  | |  |  | 20-29 |  |  |  | 30-39 |  |  |  | 40-49 |  |  |  | 50-59 |  |
| --- | --- | --- | --- | --- | --- | --- | --- | --- | --- | --- | --- | --- | --- | --- | --- | --- | --- | --- | --- | --- | --- | --- | --- | --- | --- | --- | --- | --- | --- | --- | --- | --- | --- |
|  | | | | | | | N | | | | | Mean | | | | SD | |  | N | Mean | SD |  | N | Mean | SD |  | N | Mean | SD |  | N | Mean | SD |
| Year 1996 | | | | | | |  | | | | |  | | | |  | |  |  |  |  |  |  |  |  |  |  |  |  |  |  |  |  |
| Body weight (kg) | | | | | | | 7841 | | | | | 54.68 | | | | 8.15 | |  | 2534 | 52.75 | 8.09 |  | 3102 | 54.30 | 7.50 |  | 1368 | 57.36 | 8.47 |  | 837 | 57.59 | 8.15 |
| Body height (cm) | | | | | | | 7841 | | | | | 157.46 | | | | 5.36 | |  | 2534 | 158.85 | 5.29 |  | 3102 | 157.84 | 5.11 |  | 1368 | 156.30 | 4.93 |  | 837 | 153.71 | 5.07 |
| BMI (kg/m^2^) | | | | | | | 7841 | | | | | 22.07 | | | | 3.23 | |  | 2534 | 20.90 | 3.00 |  | 3102 | 21.80 | 2.85 |  | 1368 | 23.47 | 3.24 |  | 837 | 24.36 | 3.17 |
| FPG (mg/dl) | | | | | | | 7841 | | | | | 93.64 | | | | 12.23 | |  | 2534 | 90.94 | 8.85 |  | 3102 | 92.79 | 9.31 |  | 1368 | 95.96 | 14.84 |  | 837 | 101.13 | 19.82 |
| SBP (mmHg) | | | | | | | 7841 | | | | | 111.16 | | | | 15.41 | |  | 2534 | 107.65 | 12.43 |  | 3102 | 107.96 | 12.98 |  | 1368 | 115.52 | 16.62 |  | 837 | 126.56 | 18.41 |
| DBP (mmHg) | | | | | | | 7841 | | | | | 69.32 | | | | 9.62 | |  | 2534 | 67.43 | 8.86 |  | 3102 | 68.11 | 8.98 |  | 1368 | 71.66 | 9.94 |  | 837 | 75.67 | 10.19 |
| TG (mg/dl) | | | | | | | 7841 | | | | | 87.77 | | | | 58.81 | |  | 2534 | 75.57 | 34.80 |  | 3102 | 83.70 | 48.70 |  | 1368 | 98.23 | 64.77 |  | 837 | 122.69 | 105.81 |
| HDL (mg/dl) | | | | | | | 7841 | | | | | 49.06 | | | | 12.63 | |  | 2534 | 49.07 | 12.38 |  | 3102 | 48.68 | 12.34 |  | 1368 | 49.13 | 13.11 |  | 837 | 50.38 | 13.59 |
| LDL (mg/dl) | | | | | | | 7819 | | | | | 119.96 | | | | 30.36 | |  | 2533 | 112.13 | 27.61 |  | 3095 | 117.94 | 27.74 |  | 1361 | 125.53 | 30.07 |  | 830 | 142.26 | 35.46 |
| UA (mg/dl) | | | | | | | 7841 | | | | | 5.08 | | | | 1.16 | |  | 2534 | 5.05 | 1.10 |  | 3102 | 5.00 | 1.11 |  | 1368 | 5.05 | 1.21 |  | 837 | 5.47 | 1.33 |
|  | | | | | | | N | | | | | % | | | |  | |  | N | % |  |  | N | % |  |  | N | % |  |  | N | % |  |
| Education | | | | | | |  | | | | |  | | | |  | |  |  |  |  |  |  |  |  |  |  |  |  |  |  |  |  |
| ≤ 9 year | | | | | | | 1,775 | | | | | 22.64 | | | |  | |  | 95 | 3.75 |  |  | 397 | 12.8 |  |  | 572 | 41.81 |  |  | 711 | 84.95 |  |
| 10-15 years | | | | | | | 4,767 | | | | | 60.8 | | | |  | |  | 1,902 | 75.06 |  |  | 2,133 | 68.76 |  |  | 625 | 45.69 |  |  | 107 | 12.78 |  |
| ≥ 15 years | | | | | | | 1,299 | | | | | 16.57 | | | |  | |  | 537 | 21.19 |  |  | 572 | 18.44 |  |  | 171 | 12.5 |  |  | 19 | 2.27 |  |
| Smoking | | | | | | |  | | | | |  | | | |  | |  |  |  |  |  |  |  |  |  |  |  |  |  |  |  |  |
| NA | | | | | | | 1,456 | | | | | 18.57 | | | |  | |  | 381 | 15.04 |  |  | 578 | 18.63 |  |  | 325 | 23.76 |  |  | 172 | 20.55 |  |
| Never | | | | | | | 5,870 | | | | | 74.86 | | | |  | |  | 1,927 | 76.05 |  |  | 2,307 | 74.37 |  |  | 989 | 72.3 |  |  | 647 | 77.3 |  |
| Previously | | | | | | | 82 | | | | | 1.05 | | | |  | |  | 34 | 1.34 |  |  | 40 | 1.29 |  |  | 8 | 0.58 |  |  | 5 | 0.6 |  |
| Occasionally | | | | | | | 133 | | | | | 1.7 | | | |  | |  | 54 | 2.13 |  |  | 60 | 1.93 |  |  | 14 | 1.02 |  |  | 1 | 0.12 |  |
| Frequently | | | | | | | 46 | | | | | 0.59 | | | |  | |  | 22 | 0.87 |  |  | 17 | 0.55 |  |  | 6 | 0.44 |  |  | 12 | 1.43 |  |
| Every day | | | | | | | 254 | | | | | 3.24 | | | |  | |  | 116 | 4.58 |  |  | 100 | 3.22 |  |  | 26 | 1.9 |  |  |  |  |  |
| Alcohol Drinking | | | | | | |  | | | | |  | | | |  | |  |  |  |  |  |  |  |  |  |  |  |  |  |  |  |  |
| NA | | | | | | | 952 | | | | | 12.14 | | | |  | |  | 242 | 9.55 |  |  | 386 | 12.44 |  |  | 202 | 14.77 |  |  | 122 | 14.58 |  |
| Never | | | | | | | 5,911 | | | | | 75.39 | | | |  | |  | 2,011 | 79.36 |  |  | 2,320 | 74.79 |  |  | 950 | 69.44 |  |  | 630 | 75.27 |  |
| Previously | | | | | | | 29 | | | | | 0.37 | | | |  | |  | 10 | 0.39 |  |  | 11 | 0.35 |  |  | 8 | 0.58 |  |  | 0 | 0 |  |
| Occasionally | | | | | | | 868 | | | | | 11.07 | | | |  | |  | 242 | 9.55 |  |  | 361 | 11.64 |  |  | 191 | 13.96 |  |  | 74 | 8.84 |  |
| Frequently | | | | | | | 53 | | | | | 0.68 | | | |  | |  | 20 | 0.79 |  |  | 16 | 0.52 |  |  | 11 | 0.8 |  |  | 6 | 0.72 |  |
| Every day | | | | | | | 28 | | | | | 0.36 | | | |  | |  | 9 | 0.36 |  |  | 8 | 0.26 |  |  | 6 | 0.44 |  |  | 5 | 0.6 |  |
| Use of betel nuts | | | | | | |  | | | | |  | | | |  | |  |  |  |  |  |  |  |  |  |  |  |  |  |  |  |  |
| NA | | | | | | | 495 | | | | | 6.31 | | | |  | |  | 122 | 4.81 |  |  | 206 | 6.64 |  |  | 106 | 7.75 |  |  | 61 | 7.29 |  |
| Never | | | | | | | 7,290 | | | | | 92.97 | | | |  | |  | 2,400 | 94.71 |  |  | 2,883 | 92.94 |  |  | 1,242 | 90.79 |  |  | 765 | 91.4 |  |
| Occasionally | | | | | | | 46 | | | | | 0.59 | | | |  | |  | 10 | 0.39 |  |  | 10 | 0.32 |  |  | 17 | 1.24 |  |  | 9 | 1.08 |  |
| Frequently | | | | | | | 5 | | | | | 0.06 | | | |  | |  | 1 | 0.04 |  |  | 2 | 0.06 |  |  | 1 | 0.07 |  |  | 1 | 0.12 |  |
| Every day | | | | | | | 5 | | | | | 0.06 | | | |  | |  | 1 | 0.04 |  |  | 1 | 0.03 |  |  | 2 | 0.15 |  |  | 1 | 0.12 |  |
|  | | | | | | |  | | | | |  | | | |  | |  |  |  |  |  |  |  |  |  |  |  |  |  |  |  |  |
|  | | | | | | | N | | | | | Mean | | | | SD | |  | N | Mean | SD |  | N | Mean | SD |  | N | Mean | SD |  | N | Mean | SD |
| Year 2006 | | | | | | |  | | | | |  | | | |  | |  |  |  |  |  |  |  |  |  |  |  |  |  |  |  |  |
| Body weight (kg) | | | | | | | 14259 | | | | | 54.28 | | | | 8.52 | |  | 3335 | 52.76 | 9.07 |  | 5942 | 54.06 | 8.53 |  | 3289 | 55.21 | 7.92 |  | 1693 | 56.23 | 7.85 |
| Body height (cm) | | | | | | | 14259 | | | | | 158.86 | | | | 5.42 | |  | 3335 | 160.11 | 5.27 |  | 5942 | 159.57 | 5.16 |  | 3289 | 158.02 | 5.31 |  | 1693 | 155.52 | 5.25 |
| BMI (kg/m^2^) | | | | | | | 14259 | | | | | 21.51 | | | | 3.23 | |  | 3335 | 20.57 | 3.30 |  | 5942 | 21.22 | 3.12 |  | 3289 | 22.11 | 2.98 |  | 1693 | 23.25 | 3.03 |
| FPG (mg/dl) | | | | | | | 14259 | | | | | 93.54 | | | | 11.06 | |  | 3334 | 90.34 | 6.77 |  | 5943 | 92.43 | 9.81 |  | 3289 | 95.26 | 11.01 |  | 1693 | 100.39 | 17.03 |
| SBP (mmHg) | | | | | | | 14253 | | | | | 109.59 | | | | 14.24 | |  | 3333 | 106.55 | 11.73 |  | 5939 | 107.02 | 12.24 |  | 3289 | 111.65 | 14.74 |  | 1692 | 120.61 | 17.86 |
| DBP (mmHg) | | | | | | | 14253 | | | | | 64.11 | | | | 9.63 | |  | 3333 | 61.78 | 8.19 |  | 5939 | 63.03 | 8.74 |  | 3289 | 65.45 | 10.22 |  | 1692 | 69.87 | 11.26 |
| TG (mg/dl) | | | | | | | 14259 | | | | | 79.44 | | | | 51.68 | |  | 3334 | 65.37 | 32.21 |  | 5943 | 74.94 | 42.94 |  | 3289 | 86.24 | 55.08 |  | 1693 | 109.72 | 81.37 |
| HDL (mg/dl) | | | | | | | 13541 | | | | | 59.86 | | | | 13.44 | |  | 3060 | 60.84 | 13.56 |  | 5909 | 59.62 | 13.37 |  | 3197 | 59.29 | 13.16 |  | 1675 | 59.98 | 13.88 |
| LDL (mg/dl) | | | | | | | 13539 | | | | | 111.92 | | | | 29.29 | |  | 3059 | 103.04 | 26.16 |  | 5608 | 108.56 | 26.96 |  | 3198 | 115.66 | 28.31 |  | 1674 | 132.28 | 33.14 |
| UA (mg/dl) | | | | | | | 14258 | | | | | 4.66 | | | | 1.01 | |  | 3334 | 4.67 | 0.98 |  | 5943 | 4.59 | 0.97 |  | 3289 | 4.57 | 0.99 |  | 1692 | 5.04 | 1.12 |
|  | | | | | | | N | | | | | % | | | |  | |  | N | % |  |  | N | % |  |  | N | % |  |  | N | % |  |
| Education | | | | | | |  | | | | |  | | | |  | |  |  |  |  |  |  |  |  |  |  |  |  |  |  |  |  |
| ≤ 9 year | | | | | | | 1,472 | | | | | 10.32 | | | |  | |  | 69 | 2.07 |  |  | 203 | 3.42 |  |  | 383 | 11.64 |  |  | 817 | 48.26 |  |
| 10-15 years | | | | | | | 6,637 | | | | | 46.54 | | | |  | |  | 1,157 | 34.69 |  |  | 3,037 | 51.09 |  |  | 1,848 | 56.17 |  |  | 595 | 35.14 |  |
| ≥ 15 years | | | | | | | 6,153 | | | | | 43.14 | | | |  | |  | 2,109 | 63.24 |  |  | 2,704 | 45.49 |  |  | 1,059 | 32.19 |  |  | 281 | 16.6 |  |
| Smoking | | | | | | |  | | | | |  | | | |  | |  |  |  |  |  |  |  |  |  |  |  |  |  |  |  |  |
| NA | | | | | | | 314 | | | | | 2.2 | | | |  | |  | 46 | 1.38 |  |  | 112 | 1.88 |  |  | 84 | 2.55 |  |  | 72 | 4.25 |  |
| Never | | | | | | | 12,343 | | | | | 86.54 | | | |  | |  | 2,751 | 82.49 |  |  | 5,133 | 86.36 |  |  | 2,959 | 89.94 |  |  | 1,500 | 88.6 |  |
| Secondhand | | | | | | | 669 | | | | | 4.69 | | | |  | |  | 249 | 7.47 |  |  | 264 | 4.44 |  |  | 85 | 2.58 |  |  | 71 | 4.19 |  |
| Previously | | | | | | | 160 | | | | | 1.12 | | | |  | |  | 52 | 1.56 |  |  | 76 | 1.28 |  |  | 24 | 0.73 |  |  | 8 | 0.47 |  |
| Occasionally | | | | | | | 241 | | | | | 1.69 | | | |  | |  | 70 | 2.1 |  |  | 114 | 1.92 |  |  | 48 | 1.46 |  |  | 9 | 0.53 |  |
| Every day | | | | | | | 535 | | | | | 3.75 | | | |  | |  | 167 | 5.01 |  |  | 245 | 4.12 |  |  | 90 | 2.74 |  |  | 33 | 1.95 |  |
| Alcohol drinking | | | | | | |  | | | | |  | | | |  | |  |  |  |  |  |  |  |  |  |  |  |  |  |  |  |  |
| NA | | | | | | | 531 | | | | | 3.72 | | | |  | |  | 61 | 1.83 |  |  | 61 | 1.83 |  |  | 176 | 5.35 |  |  | 139 | 8.21 |  |
| Never | | | | | | | 12,862 | | | | | 90.18 | | | |  | |  | 3,068 | 91.99 |  |  | 3,068 | 91.99 |  |  | 2,903 | 88.24 |  |  | 1,423 | 84.05 |  |
| Previously | | | | | | | 106 | | | | | 0.74 | | | |  | |  | 34 | 1.02 |  |  | 34 | 1.02 |  |  | 19 | 0.58 |  |  | 18 | 1.06 |  |
| 1-2 times/week | | | | | | | 557 | | | | | 3.91 | | | |  | |  | 144 | 4.32 |  |  | 144 | 4.32 |  |  | 143 | 4.35 |  |  | 76 | 4.49 |  |
| 3-4 times/week | | | | | | | 145 | | | | | 1.02 | | | |  | |  | 19 | 0.57 |  |  | 19 | 0.57 |  |  | 38 | 1.16 |  |  | 22 | 1.3 |  |
| Every day | | | | | | | 61 | | | | | 0.43 | | | |  | |  | 9 | 0.27 |  |  | 9 | 0.27 |  |  | 11 | 0.33 |  |  | 15 | 0.89 |  |
| Use of betel nuts | | | | | | |  | | | | |  | | | |  | |  |  |  |  |  |  |  |  |  |  |  |  |  |  |  |  |
| NA | | | | | | | 427 | | | | | 2.99 | | | |  | |  | 62 | 1.86 |  |  | 163 | 2.74 |  |  | 118 | 3.59 |  |  | 84 | 4.96 |  |
| Never | | | | | | | 13,814 | | | | | 96.86 | | | |  | |  | 3,270 | 98.05 |  |  | 5,775 | 97.16 |  |  | 3,166 | 96.23 |  |  | 1,603 | 94.68 |  |
| Previously | | | | | | | 12 | | | | | 0.08 | | | |  | |  | 3 | 0.09 |  |  | 2 | 0.03 |  |  | 4 | 0.12 |  |  | 3 | 0.18 |  |
| 1-3 times/week | | | | | | | 9 | | | | | 0.06 | | | |  | |  |  |  |  |  | 4 | 0.07 |  |  | 2 | 0.06 |  |  | 3 | 0.18 |  |
| 4-5 times/week | | | | | | |  | | | | |  | | | |  | |  |  |  |  |  |  |  |  |  |  |  |  |  |  |  |  |
| Every day | | | | | | |  | | | | |  | | | |  | |  |  |  |  |  |  |  |  |  |  |  |  |  |  |  |  |
|  |  |  |  |  |  |  | |  |  |  |  | |  |  |  | |  |  | | | | | | | | | | | | | | | |
|  |  |  |  |  |  |  | |  |  |  |  | |  |  |  | |  |  | | | | | | | | | | | | | | | |
|  |  |  |  |  |  |  | |  |  |  |  | |  |  |  | |  |  | | | | | | | | | | | | | | | |
|  |  |  |  |  |  |  | |  |  |  |  | |  |  |  | |  |  | | | | | | | | | | | | | | | |
|  |  |  |  |  |  |  | |  |  |  |  | |  |  |  | |  |  | | | | | | | | | | | | | | | |
|  |  |  |  |  |  |  | |  |  |  |  | |  |  |  | |  |  | | | | | | | | | | | | | | | |
|  |  |  |  |  |  |  | |  |  |  |  | |  |  |  | |  |  | | | | | | | | | | | | | | | |
|  |  |  |  |  |  |  | |  |  |  |  | |  |  |  | |  |  | | | | | | | | | | | | | | | |
|  |  |  |  |  |  |  | |  |  |  |  | |  |  |  | |  |  | | | | | | | | | | | | | | | |
|  |  |  |  |  |  |  | |  |  |  |  | |  |  |  | |  |  | | | | | | | | | | | | | | | |
|  |  |  |  |  |  |  | |  |  |  |  | |  |  |  | |  |  | | | | | | | | | | | | | | | |
|  |  |  |  |  |  |  | |  |  |  |  | |  |  |  | |  |  | | | | | | | | | | | | | | | |
|  |  |  |  |  |  |  | |  |  |  |  | |  |  |  | |  |  | | | | | | | | | | | | | | | |
|  |  |  |  |  |  |  | |  |  |  |  | |  |  |  | |  |  | | | | | | | | | | | | | | | |
|  |  |  |  |  |  |  | |  |  |  |  | |  |  |  | |  |  | | | | | | | | | | | | | | | |
|  |  |  |  |  |  |  | |  |  |  |  | |  |  |  | |  |  | | | | | | | | | | | | | | | |
|  |  |  |  |  |  |  | |  |  |  |  | |  |  |  | |  |  | | | | | | | | | | | | | | | |
|  |  |  |  |  |  |  | |  |  |  |  | |  |  |  | |  |  | | | | | | | | | | | | | | | |
|  |  |  |  |  |  |  | |  |  |  |  | |  |  |  | |  |  | | | | | | | | | | | | | | | |
|  |  |  |  |  |  |  | |  |  |  |  | |  |  |  | |  |  | | | | | | | | | | | | | | | |
|  |  |  |  |  |  |  | |  |  |  |  | |  |  |  | |  |  | | | | | | | | | | | | | | | |
|  |  |  |  |  |  |  | |  |  |  |  | |  |  |  | |  |  | | | | | | | | | | | | | | | |
|  |  |  |  |  |  |  | |  |  |  |  | |  |  |  | |  |  | | | | | | | | | | | | | | | |
|  |  |  |  |  |  |  | |  |  |  |  | |  |  |  | |  |  | | | | | | | | | | | | | | | |
|  |  |  |  |  |  |  | |  |  |  |  | |  |  |  | |  |  | | | | | | | | | | | | | | | |
|  |  |  |  |  |  |  | |  |  |  |  | |  |  |  | |  |  | | | | | | | | | | | | | | | |
|  |  |  |  |  |  |  | |  |  |  |  | |  |  |  | |  |  | | | | | | | | | | | | | | | |
|  |  |  |  |  |  |  | |  |  |  |  | |  |  |  | |  |  | | | | | | | | | | | | | | | |
|  |  |  |  |  |  |  | |  |  |  |  | |  |  |  | |  |  | | | | | | | | | | | | | | | |
|  |  |  |  |  |  |  | |  |  |  |  | |  |  |  | |  |  | | | | | | | | | | | | | | | |
|  |  |  |  |  |  |  | |  |  |  |  | |  |  |  | |  |  | | | | | | | | | | | | | | | |
|  |  |  |  |  |  |  | |  |  |  |  | |  |  |  | |  |  | | | | | | | | | | | | | | | |
|  |  |  |  |  |  |  | |  |  |  |  | |  |  |  | |  |  | | | | | | | | | | | | | | | |
|  |  |  |  |  |  |  | |  |  |  |  | |  |  |  | |  |  | | | | | | | | | | | | | | | |
|  |  |  |  |  |  |  | |  |  |  |  | |  |  |  | |  |  | | | | | | | | | | | | | | | |
|  |  |  |  |  |  |  | |  |  |  |  | |  |  |  | |  |  | | | | | | | | | | | | | | | |
